# Characterization of Genetic Elements Carrying *mcr-1* Gene in Escherichia coli from the Community and Hospital Settings in Vietnam

**DOI:** 10.1128/spectrum.01356-21

**Published:** 2022-02-09

**Authors:** Bich Vu Thi Ngoc, Thanh Le Viet, Mai Nguyen Thi Tuyet, Thuong Nguyen Thi Hong, Diep Nguyen Thi Ngoc, Duyet Le Van, Loan Chu Thi, Hoang Tran Huy, John Penders, Heiman Wertheim, H. Rogier van Doorn

**Affiliations:** a Oxford University Clinical Research Unit, Wellcome Africa Asia Programme, Ha Noi, Vietnam; b Quadram Institute Bioscience, Norwich, United Kingdom; c National Institute of Hygiene and Epidemiology, Ha Noi, Vietnam; d National Hospital of Tropical Diseases, Ha Noi, Vietnam; e Department of Microbiology, Hanoi Medical University, Ha Noi, Vietnam; f School for Nutrition and Translational Research in Metabolism (NUTRIM) and Care and Public Health Research Institute (Caphri), Department of Medical Microbiology, Maastricht University Medical Center, Maastricht, the Netherlands; g Department of Medical Microbiology and Radboudumc Center for Infectious Diseases, Radboud University Medical Center, Nijmegen, the Netherlands; h Center for Tropical Medicine and Global Health, Nuffield Department of Clinical Medicine, University of Oxford, Oxford, United Kingdom; University at Albany, State University of New York

**Keywords:** *mcr-1* transmission, multireplicon plasmid, plasmid harboring *mcr-1*, colistin resistance, antimicrobial resistance, One health, *Escherichia coli*

## Abstract

Colistin is widely used in agriculture and aquaculture as prophylaxis, particularly in Asia. Recently, *mcr-1* and other mobilizable genes conferring colistin resistance have spread globally in community and hospital populations. Characterizing *mcr-1* mobile genetic elements and host genetic background is important to understand the transmission of this resistance mechanism. We conducted whole-genome sequencing of 94 *mcr-1*-positive Escherichia coli isolates (Mcr1-Ec isolates) from human and animal feces, food, and water in a community cohort (N = 87) and from clinical specimens from a referral hospital (N = 7) in northern Vietnam. *mcr-1* was plasmid-borne in 71 and chromosomally carried in 25 (2 isolates contain one copy on chromosome and one copy on a plasmid) of 94 E. coli isolates from the community and hospital settings. All seven clinical isolates carried *mcr-1* on plasmids. Replicon types of *mcr-1*-carrying plasmids included IncI2, IncP, IncX4, and IncFIA single replicons and combinations of IncHI2, IncN, and IncX1 multireplicons. Alignment of a long-read sequence of an IncI2 plasmid from animal feces with short-read sequences of IncI2 plasmids from a healthy human, water, and hospitalized patients showed highly similar structures (query cover from 90% to 98%, overall identity of >81%). We detected the potential existence of multireplicon plasmids harboring *mcr-1* regardless of sample setting, confirming 10/71 with long-read sequencing. An intact/conserved *Tn6330* transposon sequence or its genetic context variants were found in 6/25 Mcr1-Ec isolates with chromosomally carried *mcr-1*. The dissemination of *mcr-1* is facilitated by a high diversity of plasmid replicon types and a high prevalence of the chromosomal *Tn6330* transposon.

**IMPORTANCE** The article presented advances our understanding of genetic elements carrying *mcr-1* in Escherichia coli in both community and hospital settings. We provide evidence to suggest that diverse plasmid types, including multireplicon plasmids, have facilitated the successful transmission of *mcr-1* in different reservoirs. The widespread use of colistin in agriculture, where a high diversity of bacteria are exposed, has allowed the selection and evolution of various transmission mechanisms that will make it a challenge to get rid of. Colocalization of *mcr-1* and other antibiotic resistance genes (ARGs) on multireplicon plasmids adds another layer of complexity to the rapid dissemination of *mcr-1* genes among community and hospital bacterial populations and to the slow pandemic of antimicrobial resistance (AMR) in general.

## INTRODUCTION

The current pandemic of antimicrobial resistance (AMR) is a major global public health challenge as countries increasingly report high rates of resistance against first- and second-line antimicrobials to treat common infections. The emergence of carbapenem resistance among *Enterobacterales* is the most urgent example. Carbapenem-resistant *Enterobacterales* are generally also resistant to other first- and second-line antibiotics and thus pose a major therapeutic challenge (1). Polymyxin B and colistin are some of the last-resort treatment options, with complex pharmacokinetics and dynamics, severe side effects, and only moderate efficacy (2, 3). The recent emergence and spread of mobile colistin resistance (*mcr*) is another major public health concern as part of the pandemic of antimicrobial resistance (4). Colistin is widely used in animal production and aquaculture, and the global spread of *mcr* genes (from *mcr-1* to *mcr-10*) among human *Enterobacterales* originated from food production animals (5, 6).

In Vietnam, colistin is commonly used in animal feed to prevent and treat animal disease and as a growth promoter (7, 8). We recently showed that *mcr*-like genes were detected in a majority of fecal samples from humans (82/93) and animals (41/45) in a rural community cohort in northern Vietnam (the Ha Nam cohort) (9).

The mobile colistin resistance (*mcr*) genes encode a member of the phosphoethanolamine transferase family that catalyzes the addition of phosphoethanolamine onto lipid A to modify lipopolysaccharide (LPS). This modification reduces the negative charge of the outer membrane of Gram-negative bacteria, conferring resistance to the polycationic colistin molecule that normally acts by disrupting the outer membrane by interacting with LPS (4, 10). The 1,602-bp *mcr-1* is usually flanked by a 765-bp putative open reading frame (ORF) encoding a protein belonging to the *PAP2* superfamily. This *mcr-1*-pap2 DNA fragment is thought to originate from porcine *Moraxella* spp., as it shares 96.5% nucleotide identity with a chromosomal *Moraxella mcr* region (11).

The rapid global dissemination of *mcr-1* is facilitated by its association with mobile genetic elements, including as a transposon (IS*Apl1-PAP2-mcr-1-*IS*Apl1*:Tn6330) and a variety of plasmids (12). Plasmid dissemination and retention among a wide range of bacterial species depend on the self-replicating potential of *mcr-1*-containing plasmids and competition with coresident incompatible plasmids. Plasmids (either with or without *mcr-1)* are generally small circular DNA structures separate from the bacterial genome that replicate independently. They are classified by replicon type (Rep) and incompatibility group (Inc) based on the sequences of essential genes for initiation and control of replication, and coresident plasmids are incompatible when they share the same replicon mechanisms (13, 14).

*Enterobacterales* isolates often coharbor *mcr-1* and other antibiotic resistance genes (ARGs) either on the same or on different plasmids. A growing number of reports show *mcr-1*-positive carbapenemase-producing *Enterobacterales* isolated from healthy animals or hospitalized patients (15, 16). Previous studies described *mcr-1* among *Enterobacterales* mainly on single-replicon plasmids, including IncX4, IncHI2, IncI2, and IncP (17, 18). Multireplicon plasmids containing *mcr-1* have been reported only rarely (19, 20). Multireplicon plasmids comprise approximately 40% of enterobacterial plasmids and are associated with mobilization and dissemination of various other antimicrobial resistance genes between different bacteria (21, 22). Incorporation of additional replicons usually expands the host range of plasmids (23). The prevalence and importance of multireplicon plasmids harboring *mcr-1* remains largely unknown.

To date, few studies have investigated the molecular characteristics of *mcr-1*-positive Escherichia coli in an entire community, including humans and their direct natural environment, where transmission of bacteria or resistance genes is possible due to exposure to common sources. Within the present study, we aimed to (i) describe the molecular characteristics of the E. coli strain harboring *mcr-1*, (ii) identify genetic elements of the plasmids carrying *mcr-1* in commensal and pathogenic E. coli, and (iii) provide the molecular features that drive the transmission of *mcr-1* in E. coli from different origins. To this end, we analyzed the whole genome and accessory genes of *mcr-1*-positive E. coli originating from humans, animals, food, water, and hospitalized patients.

## RESULTS

### E. coli harboring *mcr-1* in community setting and isolates included in whole-genome sequencing.

We found that 75% (546/725) of samples cultured on MacConkey agar with 0.5 mg/L of colistin showed growth of lactose-fermenting *Enterobacterales*, including those from feces from humans (*n* = 221/265, 83.3%) and their domestic animals (*n* = 97/122, 79.5%), water (*n* = 156/179, 87.1%), and food (*n* = 72/159, 45.2%). E. coli isolates were identified in 425 samples from human feces (*n* = 221), animal feces (*n* = 95), water (*n* = 76), and food (*n* = 33) using matrix-assisted laser desorption ionization–time of flight mass spectrometry (MALDI-TOF-MS).

DNA of E. coli isolates was subjected to PCR for *mcr-1*, and 160 isolates from 425 samples were *mcr-1* positive (Mcr1-Ec). These Mcr1-Ec isolates originated from 97 human fecal samples (*n* = 97; 36.6%) from 61 households (HHs), 42 animal stools (34.4%) from 32 HHs, 15 (8.3%) water samples from 10 HHs, and 6 (3.7%) food samples from 3 HHs. In addition, 7/140 clinical isolates were *mcr-1* positive by PCR. A total of 160 Mcr1-Ec isolates in the community cohort and all 7 clinical isolates were further used for antimicrobial susceptibility testing.

**FIG 1:**
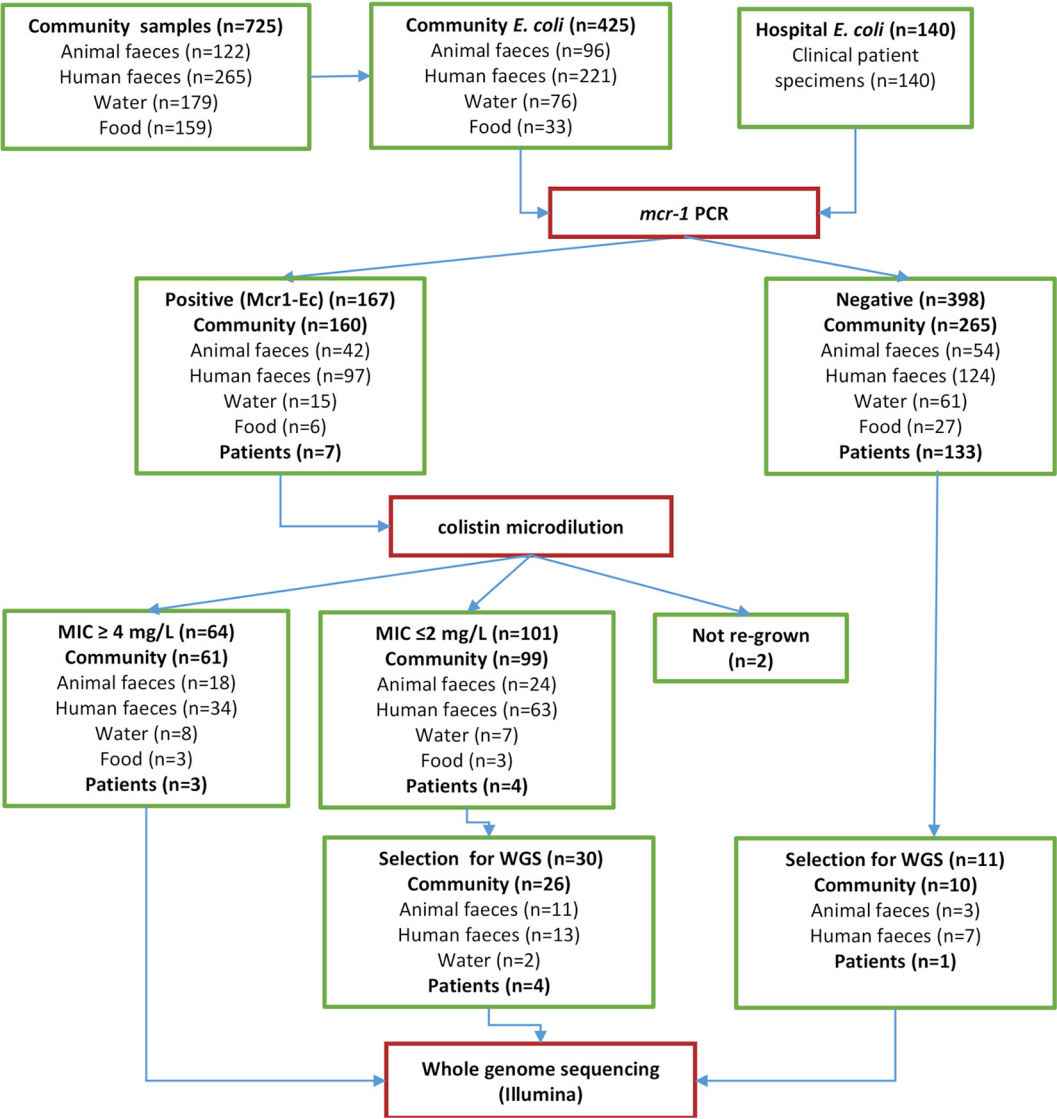
A flowchart of experimental design and sample selection for whole-genome sequencing in this study.

Colistin broth microdilution testing showed that 97/160 (58.8%) community cohort Mcr1-Ec isolates were phenotypically susceptible to colistin with an MIC of ≤2 mg/L. We excluded two isolates that failed to grow from further analysis. The remaining 61 isolates were resistant to colistin with MIC values between 4 and ≥16 mg/L (Fig. 1). Among the clinical isolates, 3/7 were phenotypically resistant to colistin (blood culture [*n* = 1] and throat swab [*n* = 2]).

A total of 105 isolates were selected for whole-genome sequencing: 94 Mcr1-Ec isolates (all clinical isolates [*n* = 7], all community cohort isolates with colistin MIC of ≥4 mg/L [*n* = 66], and 21 randomly selected susceptible Mcr1-Ec isolates) and 11 *mcr-1*-negative control isolates as the control for whole-genome sequencing. (Fig. 1).

### Genetic characterization of E. coli harboring *mcr-1*.

**FIG 2:**
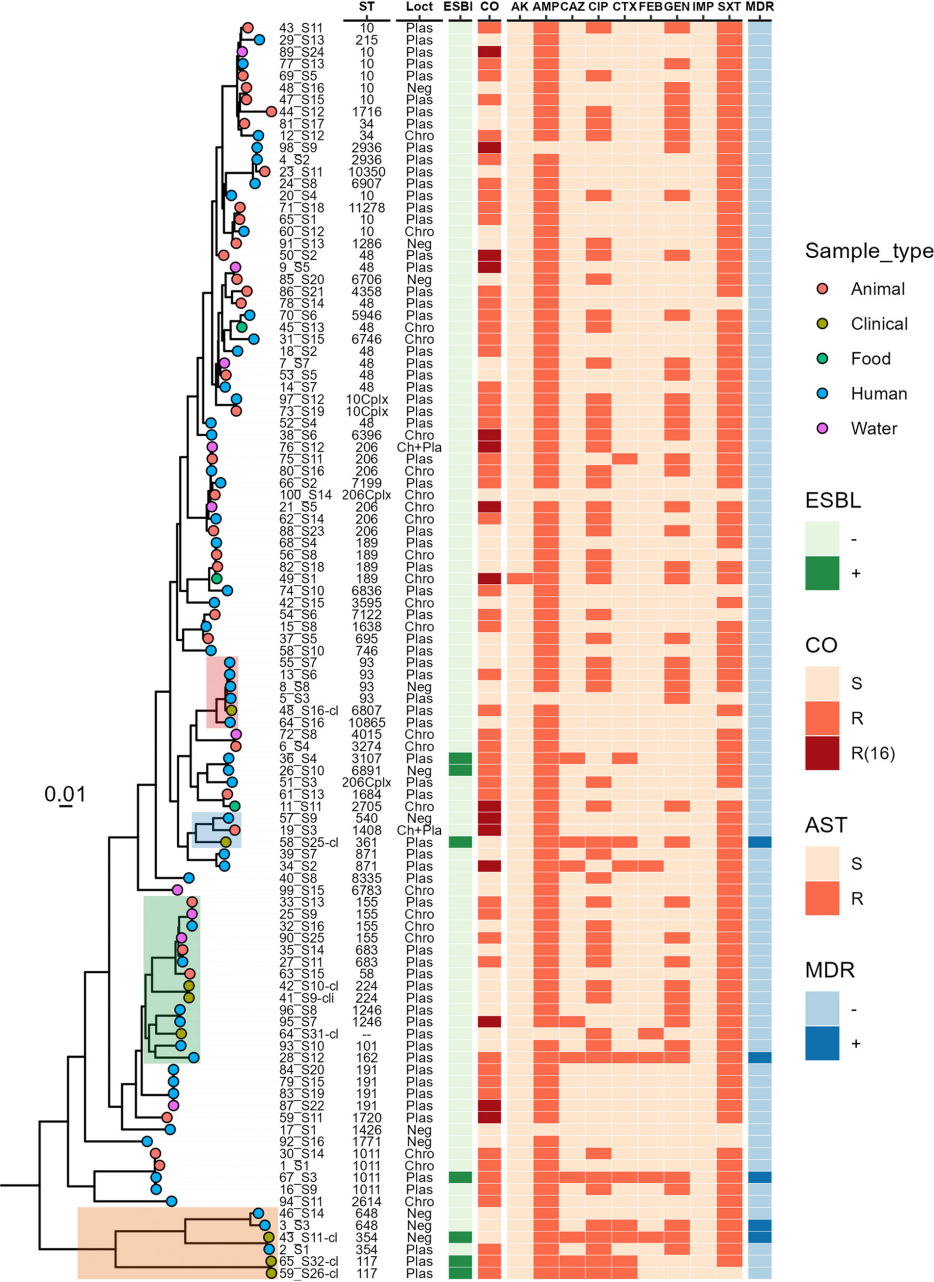
Genotypes and phenotype of E. coli isolates from different origin. From left to right. Phylogenetic tree, constructed by maximum-likelihood phylogeny. E. coli isolates from clinical samples reside on 4 branches (colored) with E. coli isolates from human feces from the community cohort. ST column indicates the sequence type of isolates, as determined by MLST, Loct column indicates the presence of *mcr-1* on chromosome and/or plasmid. Green boxes in ESBL column indicate presence of ESBL genes. The heatmap represents the antimicrobial resistance phenotype of 94 Mcr1-Ec and 10 *mcr-1*-negative E. coli isolates from different origins, orange boxes indicate phenotypic resistance (R), white boxes indicate phenotypic susceptibility (S). The column CO indicates colistin susceptibility of isolates, red boxes (R16) indicate MIC values of colistin ≥16 mg/L, orange boxes (R) indicate MIC values of colistin between 4 to 8 mg/L, and light orange boxes indicate MIC values of colistin ≤2 mg/L. The column MDR indicates multi drug resistance of isolates.

Whole-genome sequencing (WGS) identified a high diversity of multilocus sequence types (MLST) among Mcr1-Ec isolates: 93/94 Mcr1-Ec isolates belonged to 57 different sequence types (STs). One clinical isolate was not typed but shared 6/7 alleles with ST2702. The most common STs included ST10 (*n* = 10, 11%), ST48 (*n* = 9, 9%), and ST206 (*n* = 8, 8%). The STs of clinical Mcr1-Ec isoilates varied: ST234 (*n* = 2), ST117 (*n* = 2), ST361 (*n* = 1), ST6807 (*n* = 1), and ST2702-like (*n* = 1). None of the clinical STs was found in the community cohort (Fig. 2 and supplemental material).

A phylogenetic tree of Mcr1-Ec (*n* = 94) and control isolates (*n* = 11) was constructed based on 108,184 core genome single nucleotide polymorphisms (SNPs). The core gene phylogeny revealed that with the exception of a few branches that contained only human commensal and clinical isolates, no clear clustering of isolates based on sample type was observed. However, our clinical isolates cluster only with our human commensal isolates but not with other sample types. Human commensal isolates, on the other hand, cluster together with isolates from all kinds of sample types, suggesting a coalescence between reservoirs (Fig. 2).

### Resistome of E. coli harboring *mcr-1*.

We detected a total of 57 unique ARGs conferring resistance to 16 distinct antibiotic classes. Among 94 Mcr1-Ec isolates, 89 isolates contained at least one gene conferring resistance to beta-lactams (Table S1), 80 of which were phenotypically resistant to AMP. Four variants of *bla*_CTX-M_ genes encoding extended-spectrum β-lactamases (ESBLs) were found infrequently among 6/94 (6%) isolates: 14, 15, 27, and 55. Four of these isolates had a clinical origin. No phenotypic resistance against carbapenems or carbapenem resistance genes were detected (Fig. 2). Proportions of genes conferring resistance to cotrimoxazole, fluoroquinolones, and aminoglycosides are shown in Table S1.

### Characterization of chromosomal and plasmid-borne *mcr-1* using short-read sequencing.

*mcr-1* from all (*n* = 94) Mcr1-Ec isolates was 100% identical to published *mcr-1.1* sequences (accession no: KP347127). *mcr-1* from 25/94 (26%) isolates was located chromosomally, and 6 of these were phenotypically susceptible. No chromosomal *mcr-1* was found among clinical isolates. Notably, we detected two isolates containing two copies of *mcr-1*, one on the chromosome and one on a plasmid; both had a colistin MIC of >16 mg/L. All remaining isolates (*n* = 69) had *mcr-1* detected only on plasmids.

*In silico* typing of *mcr-1*-carrying plasmids from 71 isolates based on short-read sequencing data showed 29 single-replicon plasmids belonging to 7 types among 29 isolates, including IncI2 (*n* = 9, 13%), IncP (*n* = 7, 10%), InX4 (*n* = 5, 7.5%), IncFIA (*n* = 3, 4.5%), IncHI1B (*n* = 3, 4.5%), IncN (*n* = 1), and IncX1 (*n* = 1). In addition, 24 different multireplicon plasmid types were found among 36 isolates, with combinations of IncFIA, IncFIB, IncHI1B, IncHI2, IncN, IncY, IncX1, and IncI2. The most common combinations were between IncHI2 with other replicon types (*n* = 23, 64%) and IncH with IncF (*n* = 12, 33%). A total of 13/31 replicon types were found in both humans and animals (Table 1 and supplemental material, *In silico mcr-1* plasmids).

**TABLE 1:** *In silico* molecular plasmids harboring *mcr-1*^*a*^

Replicon_formula (*in silico*)	No. of isolates (short read)	Predicted size (Kb)	Predicted mobilizable	No. of ARGs	No. of isolates (long read)	Replicon_formula (long-read sequencing)	Source(s)
FIA	3	150–300	Conjugative	9			Ani_Hu
FIA: FIC: rep2327	1	50–100	Nonmobilizable	6			Ani
FIA: HI2A: HI2	1	>300	Conjugative	15	1	FIA: FIB: HI2A: R: X1: HI2	Cli
FIB: FIC: rep2244: HI2A: HI2	1	200–300	Conjugative	14	1	FIB: FIC: rep2244: HI2A: HI2	Cli
FIB: FIC: rep2244: HI2A: N	1	200–300	Conjugative	6			Cli
FIB: HI1B	2	100–150	Nonmobilizable	11			Ani-Wa
FIB: HI1B: HI1B	2	150–200	Mobilizable	3			Ani_Hu
FIB: HI1B: HI1B: N	1	100–150	Mobilizable	2			Ani
FIB: HI1B: HI2A: HI2: N	1	>300	Mobilizable	10			Hu
FIB: HI1B: N	2	50–150	Nonmobilizable	4			Ani_Hu
FIB: I2	1	<50	Nonmobilizable	0			Hu
FIC: rep2244: HI2A: HI2	1	>300	Conjugative	8			Hu
HI1B	3	50–100	Mobilizable	7			Wa-Hu
HI1B: HI1B	1	100–150	Nonmobilizable	4			Hu
HI1B: HI1B: rep2327	1	50–100	Mobilizable	2			Hu
HI1B: HI2A: HI2	2	200–300	Mobilizable	16	1	HI2A: HI2	Ani-Cli
HI1B: HI2A: HI2: N	4	>300	Conjugative	20	2	HI2A: HI2: N	Ani_Hu
HI1B: X1	2	100–150	Nonmobilizable	9			Ani_Hu
HI1B: X2	1	100–150	Mobilizable	6			Hu
HI1B: rep2327	1	100–150	Nonmobilizable	2			Ani
HI2A: HI2	3	200–300	Conjugative	17	1	HI2	Ani_Hu
HI2A: HI2: I2	2	>300	Conjugative	13			Hu
HI2A: HI2: N	1	200–300	Conjugative	8			Water
HI2A: HI2: Y	2	200–300	Conjugative	15			Ani_Hu
HI2A: I2: N: HI2	1	200–300	Conjugative	9			Hu
HI2A: N	1	150–200	Conjugative	2			Cli
I2	9	50–100	Conjugative, non-mobilizable	1	2	I2	Ani_Hu_Wa_Cli
N	1	50–100	Nonmobilizable	7			Ani
P	7	50–100	Conjugative, mobilizable	1	1	P	Ani_Hu
X1	1	<50	Nonmobilizable	6			Hu
X4	5	<50	Conjugative	3			Ani_Hu
Undetected	6	<50	Conjugative, nonmobilizable	0			Ani_Hu

a The table represents a list of replicon plasmid types in Mcr1-Ec, according to their molecular characteristics, as determined by PlasmidFinder. Column source indicates the origin of plasmids (from humans [Hu], animals [Ani], water [Wa], clinical samples [Cli]).

Among single-replicon plasmids, *mcr-1* genes were detected in the same contigs as replicons in eight Mcr1-Ec isolates of community origin, including IncI2 (*n* = 3), IncP (*n* = 2), InX4 (*n* = 2), and IncHI1B (*n* = 1) (supplemental material, *In silico mcr-1* plasmids). Multireplicon types belonging to IncHIA/IncHI1B (*n* = 1) and IncHI2/IncHI2A (*n* = 1) were also found on the same contigs as *mcr-1*. Among seven Mcr1-Ec isolates of clinical origin, five isolates carried more than one Inc-type, including IncHI2 (*n* = 4), IncFIA (*n* = 3), and IncFIB (*n* = 3). However, these were not found on the same contigs and we cannot conclude whether these were multi- or single-replicon plasmids.

Among *in silico* plasmids in Mcr1-Ec isolates, the plasmid size varied among replicon types, ranging from 10 kp to 300 kp (Table 1). A total of 26/29 of *in silico* single-replicon plasmids, including IncI2, IncP, IncX, and IncHI1B, were found in both humans and animals and were smaller than 100 kb. Regarding the ARG profile of *in silico* plasmids carrying *mcr-1*, a median number of 9 (range 1 to 17) ARGs were found to be located on the same *in silico* plasmid as *mcr-1* (Table 1). A total of 22/71 *in silico* plasmids with only *mcr-1* included the following replicon types: IncI2 (*n* = 7), IncP (*n* = 6), IncX4 (*n* = 3), IncHI1B (*n* = 1), Inc(FIB:HI1B:N) (*n* = 1), Inc (FIB:I2) (*n* = 1), and an unknown replicon type (*n* = 3). We did not find a significant association between plasmid replicon types and colistin resistance phenotype (*P* value of >0.05). However, we observed that among 22 isolates carrying plasmids with only *mcr-1*, 15 isolates had a colistin-resistant phenotype (MIC values of 4 to 16 mg/L). Additionally, we observed that 15 of 94 sequenced isolates were resistant to colistin with an MIC of ≥16 mg/L (Fig. 2). Among these 15 isolates, 2 isolates contained two copies of *mcr-1* (one located on the chromosome and the other on a plasmid: IncP and IncI2), followed by plasmid-borne (*n* = 7) and *mcr-1* chromosomal isolates (*n* = 4). No clinical isolate was resistant to colistin at an MIC of ≥16 mg/L.

### Confirmation of *in silico* multireplicon plasmids and Tn6630 structure by long reads.

To further characterize the structure of the *mcr-1*-carrying elements, we sequenced 10 isolates representing 6 replicon types using the MinION (Oxford Nanopore Technologies) nanopore sequencer. Long-read results confirmed the findings of *in silico* short-read results (Table 1 and supplemental material, Plasmid long & short read), including 3 single-replicon types, IncI2 (*n* = 2), IncP (*n* = 1), InHI2 (*n* = 1), and 3 multireplicon types, IncHI2 and IncHI2A (*n* = 6) from community (*n* = 4) and clinical Mcr1-Ec origin (*n* = 2). Nanopore sequencing also detected *mcr-1* on the chromosome of Mcr1-Ec (*n* = 2). In addition to this confirmation, the long-read data showed the presence of IncN (*n* = 2) and IncX1/IncR (*n* = 1) replicon types on the contigs containing IncHI2 and IncHI2A of clinical origin. These data enabled us to construct two putatively circular multireplicon plasmids harboring *mcr-1* (supplemental material). Notably, a total of five multireplicon plasmids, which originated from hospitalized patients (*n* = 2) and community humans (*n* = 3), coharbored bla_CTX-M_ and *mcr-1*.

**FIG 3:**
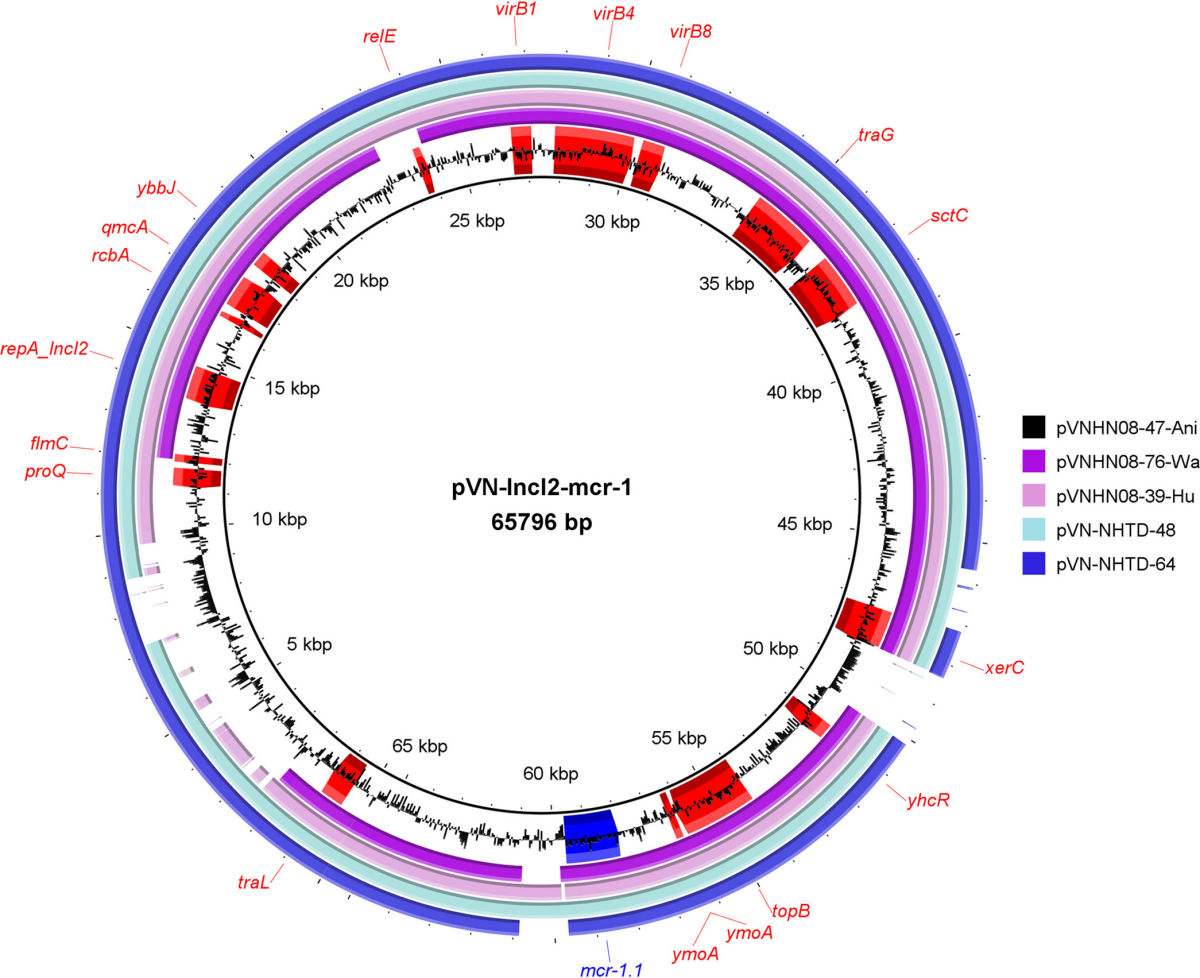
Using polishing long reads and short reads to compare sequence of IncI2 plasmids from human and animal feces and water in the same habitat and from clinical samples, BRIG visualizes a comparison of IncI2 plasmids from community cohort (pVNHN08-47-Ani [black] from animal feces, pVNHN08-76-Wa [purple] from water, pVNHN08-39-Hu [violet] from human feces) with IncI2 plasmids in the hospital setting (pVN-NHTD-48 and pVN-NHTD-64).

Long-read sequences of IncI2 carrying *mcr-1* from animal feces were similar to those of *in silico* IncI2 plasmids from humans (query cover: 90%) and hospitalized patients (query cover: 98%) with identity ranging from 81% to 100% (Fig. 3). In addition, we observed a query cover at 100% and no sequence gaps in a comparison of two long-read sequences of a multireplicon plasmid Inc(HI2:HI2A:N) from human and animal feces (Fig. S1). These results suggest that the presence of plasmids harboring *mcr-1* in E. coli might be a result of a horizontal gene transfer between microbiota of animals, water, humans, and patients (Fig. 3).

**FIG 4:**
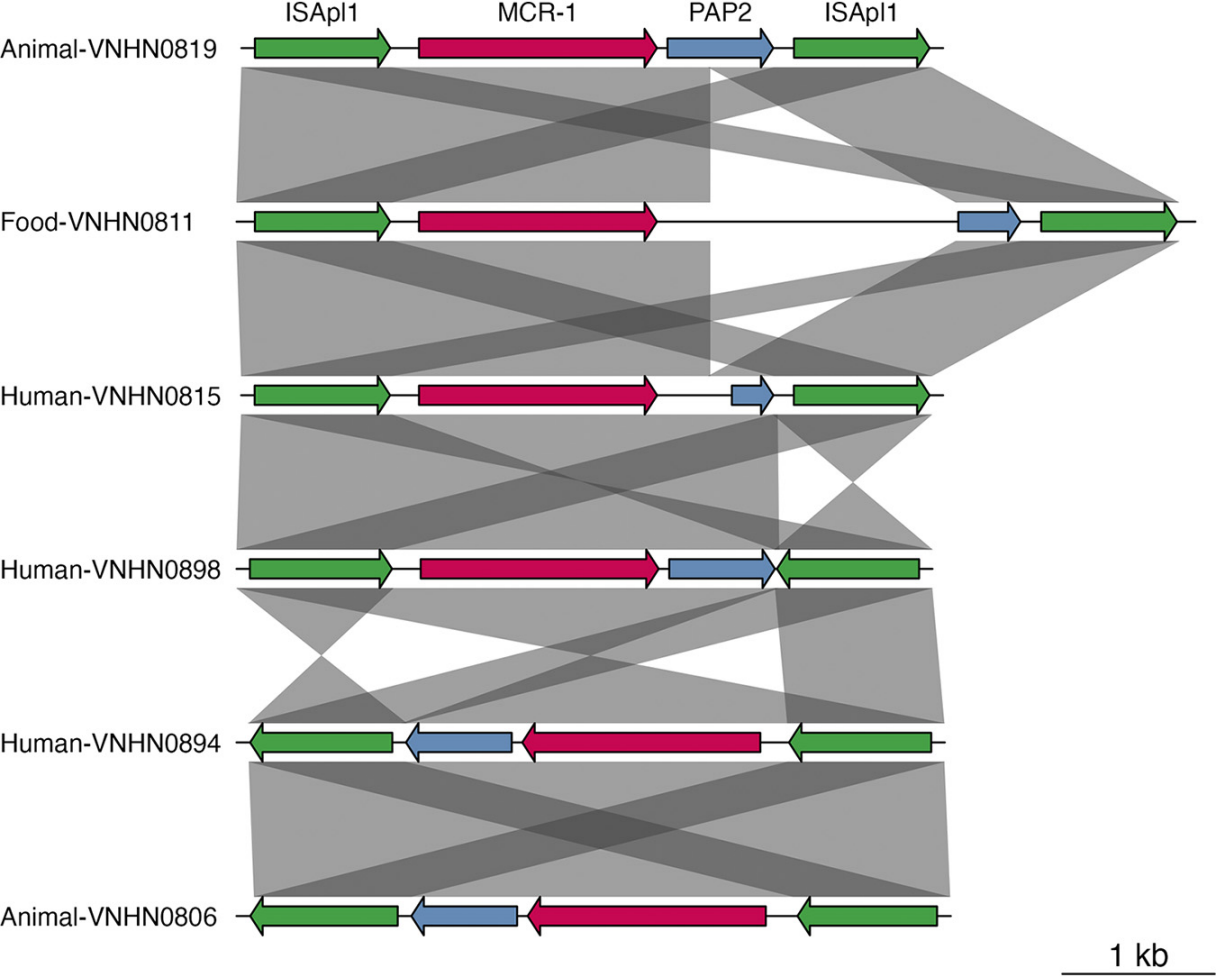
Six representative sequences showing the structural similarity between composite transposon *Tn6330* identified on chromosome of Mcr1-Ec from humans, animals, and food in the study cohort. *ISApl1* transposase, long green arrow; *mcr-1*, long pink arrow; pap2, blue arrow.

We generated a second hybrid assembly using the data of both Illumina and Nanopore platforms for 10 (10%) Mcr1-Ec isolates. Six different genetic contexts of *mcr-1* with presence/absence of insertion sequence *ISApl1* were found on both chromosomes and plasmids. Among isolates carrying *mcr-1* on their chromosome (*n* = 6), five isolates contained the full *Tn6330* transposon sequence *ISApl1-pap2-mcr-1-ISApl1*. The one remaining isolate had a combination of *ISApl1* and *IS91* (*ISApl1-mcr-1-IS91*) (Fig. 4). Two single-ended variants (*ISApl1* and *IS1A*) were found in two multireplicon Inc(HI2:HI2A) and Inc(HI2:HI2A:N) plasmids (pVNHN08-95 and pVNHN08-84, respectively). No full transposon was found on plasmids. The Mcr1-Ec isolate (VNHN08-19) that carried two copies of *mcr-1* had one chromosomal *Tn6330* transposon and one *mcr-1* copy on IncP plasmid.

## DISCUSSION

Bacteria carrying colistin resistance genes on plasmids are found worldwide in a wide range of hosts and reservoirs, and new *mcr-1*-like genes continue to be detected (*mcr-2* to *mcr-10*) (24). Previous studies have revealed which plasmid types are involved in dissemination of *mcr-1* due to horizontal gene transfer in various environments like the animal gut (25). Here, we characterize the core genome and accessory genes of E. coli harboring *mcr-1* genes in a community cohort and hospitalized patients.

Retrospective studies have demonstrated that *mcr-1* has been detected in isolates from 2005 onward, mostly in isolates from pigs and chickens (18, 26). Previous studies also showed that the proportion of *mcr-1* carriage among human clinical isolates was less than 1% (27). This proportion was 5% (7/140) among clinical isolates in this study. Recently, coexistence of *mcr-1* and genes encoding carbapenemases in different reservoirs has been sporadically reported (28, 29), but it was not observed in this study. The relatively low proportion of Mcr1-Ec in hospital settings in this and other studies may reflect the limited use of colistin in clinical practice as opposed to the higher use of colistin in agriculture with corresponding higher proportions of resistant isolates among food-production animals and agricultural communities.

Recent studies reported that E. coli isolates carrying *mcr-1* are often phenotypically susceptible or resistant to colistin at low MIC values (<16 mg/L) (30). In this study, we used a relatively low concentration of colistin (0.5 μg/mL) that both inhibits growth of naturally susceptible species such as *Pseudomonas* and *Acinetobacter* and allows for selection of *mcr-1*-carrying *Enterobacterales*. The exact mechanism of *mcr-1* gene expression in relation to resistance is still unclear (31). Our study showed that two isolates with two copies of *mcr-1* (one on the chromosome and one on a plasmid) were resistant to colistin at an MIC value of over 16 mg/L. Despite absence of evidence of an association between MIC values and *mcr-1* copy number or location, the prevalence of Mcr1-Ec isolates with a high MIC value of colistin in a community setting and the possible exchange of mobile elements between community and clinical isolates could present a clinical challenge in hospital settings in the future.

In line with earlier studies, single-replicon plasmids harboring *mcr-1* were mostly IncI2, IncP, IncX4, and IncHI2 in E. coli. This study showed that those plasmids harboring *mcr-1* were found in both the animal and human reservoirs. Indeed, the high sequence identity between IncI2 plasmids of community and clinical origin Mcr1-Ec supports the possibility of horizontal transmission of this plasmid from an environmental to a clinical E. coli strain. This supports the hypothesis that the emergence and transmission of *mcr-1* were a consequence of frequent use of colistin in animal production for food and that *mcr-1* was successfully transmitted to humans through various plasmid types.

By confirming short-read and long-read data, we show the presence of multireplicon plasmids harboring *mcr-1* as a result of incorporation of IncHI2 and other replicons (IncN, IncX, and RepA_2244). The colocalization of two replicons forms a multireplicon plasmid, in which one replicon drives the plasmid replication due to selective pressure of high use of antibiotics while the other one freely evolves through sequence divergence to cross over phylogenetic barriers between bacterial species (32). For instance, IncHI1/IncHI2 plasmids often carry a cassette of ARGs conferring resistance to colistin, sulfonamides, aminoglycosides, tetracycline, and streptomycin. Meanwhile, IncN plasmids are found most widely in bacteria of human and animal origin (33). In addition, the multireplicon status allows plasmids to avoid replacement or expulsion by incoming incompatible plasmids and to maintain their genetic characteristics in next generations of the host bacteria (34). Therefore, the existence of multireplicon plasmids harboring *mcr-1* provides an explanation for the successful onward transmission and dissemination of these plasmids and the high prevalence of *mcr-1* in feces from human and animal origins in our studies (9).

Community isolates with chromosomal *mcr-1* carried the complete ancestral transposon IS*Apl1*-*mcr-1*-*pap2*-IS*Apl1* (*Tn6330*), as has been described by others (12). Like other ARGs, once successfully integrated in the genome, the integrated *mcr-1* will slowly acquire the characteristics of host genome (amelioration) while losing the ancestral structure (35). Thereby, it will increase its stability and may change its expression level or function (35, 36). As a result, the stability of chromosomal insertion will ensure vertical transmission of *mcr-1*. Our study did not find the *Tn6330* without its *ISApl1* flanking sequence in chromosome of Mcr1-Ec, suggesting relatively recent insertion.

In this present study, the combination of short-read and long-read sequencing approaches enabled us to predict full genome and plasmid structure with high accuracy. We conducted a comprehensive genetic analysis of *mcr-1* carriers in order to have a better understanding of the prevalence and genetic background of *mcr-1*. A limitation of this study is that the assessed samples may not broadly represent Mcr1-Ec in Vietnam, as we collected samples in only one rural cohort in northern Vietnam.

In conclusion, in this study, we showed the widespread occurrence of *mcr-1* genes and colistin resistance in Vietnamese community (2015 to 2016) and hospital settings (2016 to 2017) and we characterized the genetic background of *mcr-1* in E. coli. Though E. coli isolates from clinical and community settings vary, these data suggest that similar genetic elements are shared between these settings. The widespread use of colistin in agriculture, where a high diversity of bacteria are exposed, has allowed selection and evolution of various transmission mechanisms that will make it a challenge to get rid of. Colocalization of *mcr-1* and other ARGs on multireplicon plasmids adds another layer of complexity to the rapid dissemination of *mcr-1* genes among community and hospital bacterial populations and to the slow pandemic of AMR in general.

## MATERIALS AND METHODS

### Sample collection and bacterial culture.

Samples were collected in the Ha Nam household cohort in Ha Nam, Vietnam (37). This study was part of a previously described longitudinal study (November 2015 to April 2016) to assess the microbiome and resistome of humans and their food, animals, and water (9). Here, we used a cross-sectional sample taken at month 2 including feces from humans (*n* = 265) and their domestic animals (dogs, chickens, pigs, buffaloes, and cows) (*n* = 122), food (meat and vegetables) (*n* = 159), and water samples (*n* = 179).

Feces from humans and animals were inoculated on MacConkey agar (Oxoid, UK) containing 0.5 μg/mL of colistin (Sigma). Food and water samples were preprocessed as described previously (9). Briefly, 100 mL of water was filtered through a membrane with 0.45-μm pore size. One quarter of the membrane was inoculated in tryptic soy broth at 37°C. Two grams of processed food was cut into pieces of ∼2 mm and enriched overnight in tryptic soy broth at 37°C without shaking. We used 10 μL of enrichment broth of each sample for culture on MacConkey agar containing 0.5 μg/mL of colistin. For each sample, up to 5 large pink colonies with different morphologies were picked (38). We subcultured all isolates on nutrient agar (Sigma) without colistin for further analysis. Isolates were identified using MALDI-TOF MS (Bruker, Berlin, Germany). All E. coli isolates were selected for further screening for *mcr-1*.

To investigate the transmission between community and hospital settings, we also included all E. coli isolates (*n* = 140) obtained from specimens from patients hospitalized at the National Hospital for Tropical Diseases (NHTD), Hanoi between June 2016 and October 2017.

DNA of E. coli isolates was subjected to PCR to detect the *mcr-1* gene as described previously (4).

### Antimicrobial susceptibility testing.

The minimum inhibition concentration (MIC) of colistin (CO) was determined using broth microdilution. The interpretation of colistin susceptibility was based on the breakpoint value defined by EUCAST (39). All *mcr-1*-positive E. coli isolates (Mcr1-Ec isolates) were tested for antibiotic susceptibility by agar microdilution for the other antibiotics. The panel of antibiotics included ampicillin (AMP), ceftazidime (CAZ), cefotaxime (CTX), cefepime (FEB), gentamicin (GEN), ciprofloxacin (CIP), trimethoprim-sulfamethoxazole (SXT), amikacin (AK), imipenem (IMP), and meropenem (MEM). Antimicrobial resistance of isolates was determined using the breakpoint criteria from the Clinical and Laboratory Standards Institute (40). We deducted multidrug resistance (MDR) status using a definition of resistance against third-generation cephalosporins, aminoglycosides, and fluoroquinolones while remaining susceptible against carbapenems (41).

### Whole-genome sequencing.

We sequenced all resistant *mcr-1*-positive E. coli isolates and randomly selected susceptible and *mcr-1*-negative isolates using randomizer (www.randomizer.org). We prepared the Illumina libraries using the Nextera XT kit (Illumina, San Diego, CA, United States). Paired-end 150-bp reads on fragments of 300-bp insert size were sequenced on a Miseq platform. We performed *de novo* assembly of samples using Shovill v1.1.0 with SPAdes v3.14.1 as the assembler (42). NCBI Prokaryotic Genome Annotation Pipeline (PGAP) was used to annotate genes (43). ABRicate (for detection of AMR genes from ResFinder databases of the Center for Genomic Epidemiology) and Staramr (scans genome contigs against ResFinder. PlasmidFinder databases, https://libraries.io/pypi/staramr) were used to find ARGs, and MLST was used to identify multilocus sequence type for the samples.

The core genome was generated with Prokka (44) and Roary (45) and fed into IQ-TREE v1.6.11 (46) to reconstruct a maximum-likelihood phylogenetic tree. The R graphic packages, including ggtree and ggplot2, were used for visualizing the results (47).

In order to reconstruct full circular plasmid sequences with numerous repetitive elements, we also conducted sequencing using Oxford Nanopore technology. Based on the *in silico* results of analysis of the short-read data, we selected isolates representing replicon types located on plasmids harboring *mcr-1*, regardless of origin. We prepared the libraries for ONT with the kit SQK-LSK108 following the ONT protocol and a flow-cell version FLO-MIN106 R9.5. The pool was then sequenced for 24 h on a MinION device. The raw data in fast5 format were base called with the high-accuracy mode and demultiplexed using Guppy 4.2.2 (48). We reconstructed the sample genomes by employing a hybrid *de novo* assembly approach. This process included assembling the long reads with Flye v2.8 (49) and following a 5-round of polishing using Pilon (50) with the Illumina short reads of the same sample. We visualized the comparisons of the complete E. coli chromosomes and plasmids harboring *mcr*-1 using BRIG (51).

### Data availability.

The data for this study have been deposited in the European Nucleotide Archive (ENA) at EMBL-EBI under accession number PRJEB47011 (https://www.ebi.ac.uk/ena/browser/view/PRJEB47011). Accession numbers have been listed in the accession number sheet in the supplemental material. MLST profiles were submitted on https://PubMLST.org with the submission number BIGSdb_20211018084107_007367_32640.
