# Supplementary material for: Characterization of Genetic Elements Carrying mcr-1 Gene in Escherichia coli from the Community and Hospital Settings in Vietnam
Source: Microbiol Spectr. 2022 Feb 9;10(1):e01356-21. doi: 10.1128/spectrum.01356-21 (PMC8826730; doi:10.1128/spectrum.01356-21)
Supplement: SUPPLEMENTAL FILE 2 — Supplemental material. Download SPECTRUM01356-21_Supp_1_seq9.pdf, PDF file, 0.7 MB [file spectrum01356-21_supp_1_seq9.pdf]

Characterization of genetic elements carrying *mcr-1* gene in *Escherichia coli* from the community and hospital setting in Vietnam

Vu Thi Ngoc Bich[1, 7], Le Viet Thanh[2], Nguyen Thi Tuyet Mai[3], Nguyen Thi Hong Thuong[1], Nguyen Thi Ngoc Diep[1], Le Van Duyet[4], Chu Thi Loan[5], Tran Huy Hoang[3], John Penders[6], Heiman Wertheim[7, 8], H. Rogier van Doorn[1, 8].

Supplementary Figures

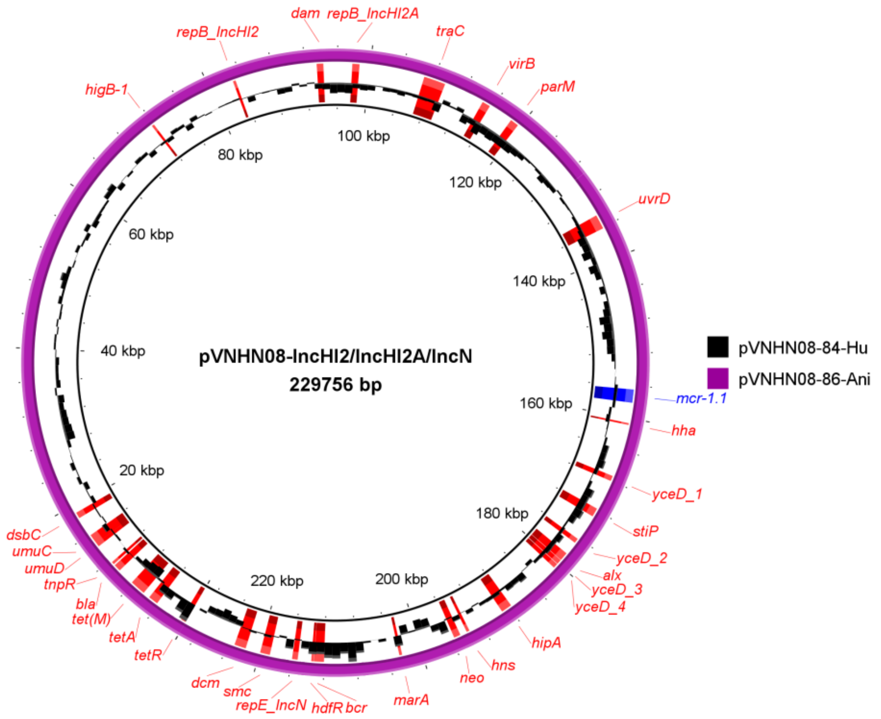

Figure S1: BRIG visualize a comparison between multi replicon plasmids, pVNHN08-84-Hu, pVNHN08-86-Ani from human faeces and domestic animal faeces, respectively. The label in the outer ring represent the annotation on the genes associates to antibiotic resistance genes, insertion elements.

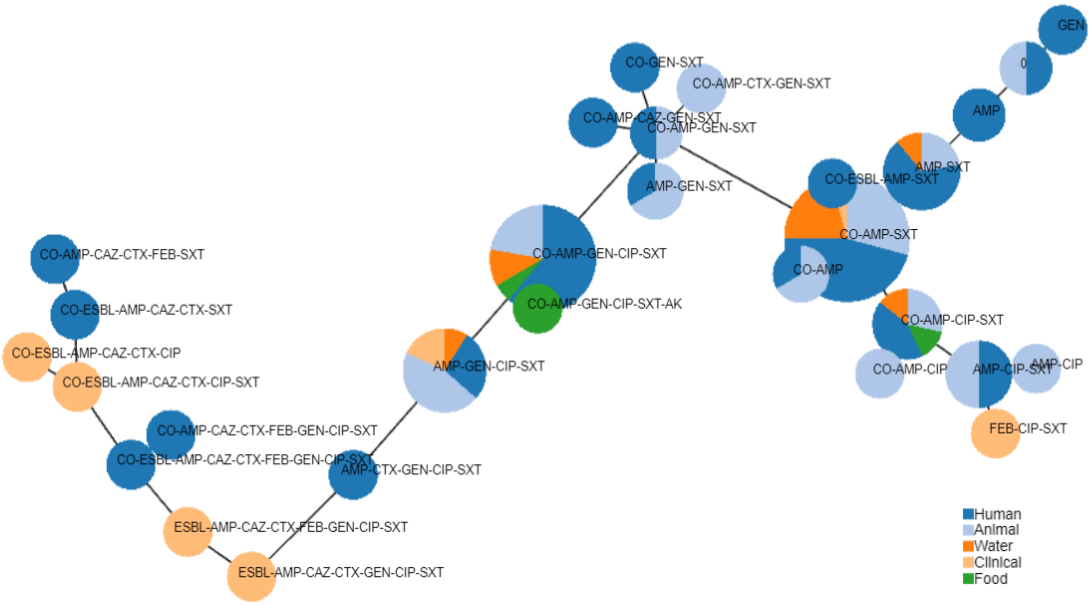

Figure S2: Minimum spanning tree showing the antimicrobial resistance (AMR) profiles representing 94 Mcr1-Ec isolates from community and hospital settings in Vietnam. Each circle represents a unique AMR profile; its size is directly proportional to the number of isolates with that profile. The colours indicates the origination of the host.

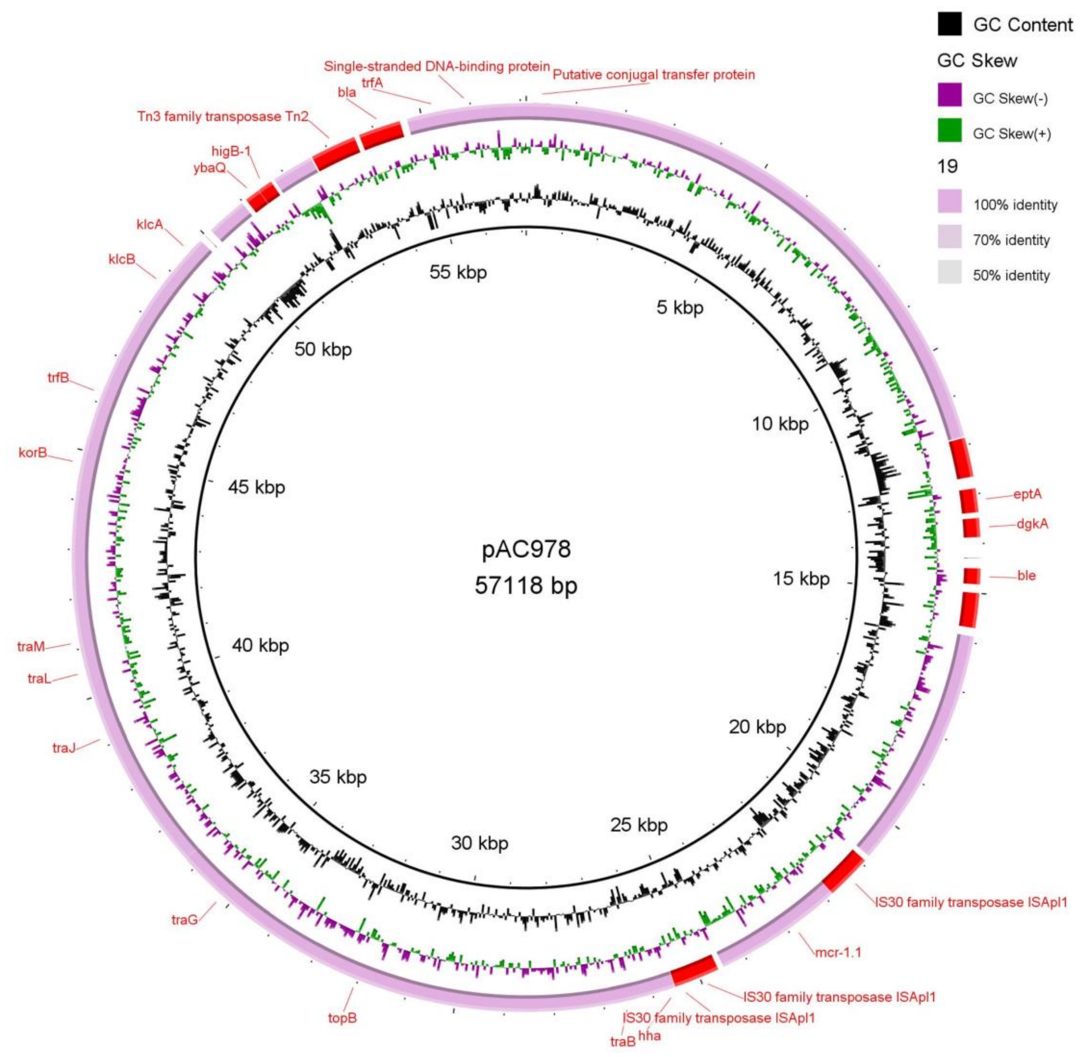

Figure S3: Using polishing long reads and short reads to construct the plasmid map. BRIG visualization a comparison of pVNHN08-19 (purple), pVNHN08-76 (blue) and pVNHN08-41 (light blue) in this study and reference plasmid pMCR-1511 (Accession number: KX377410).

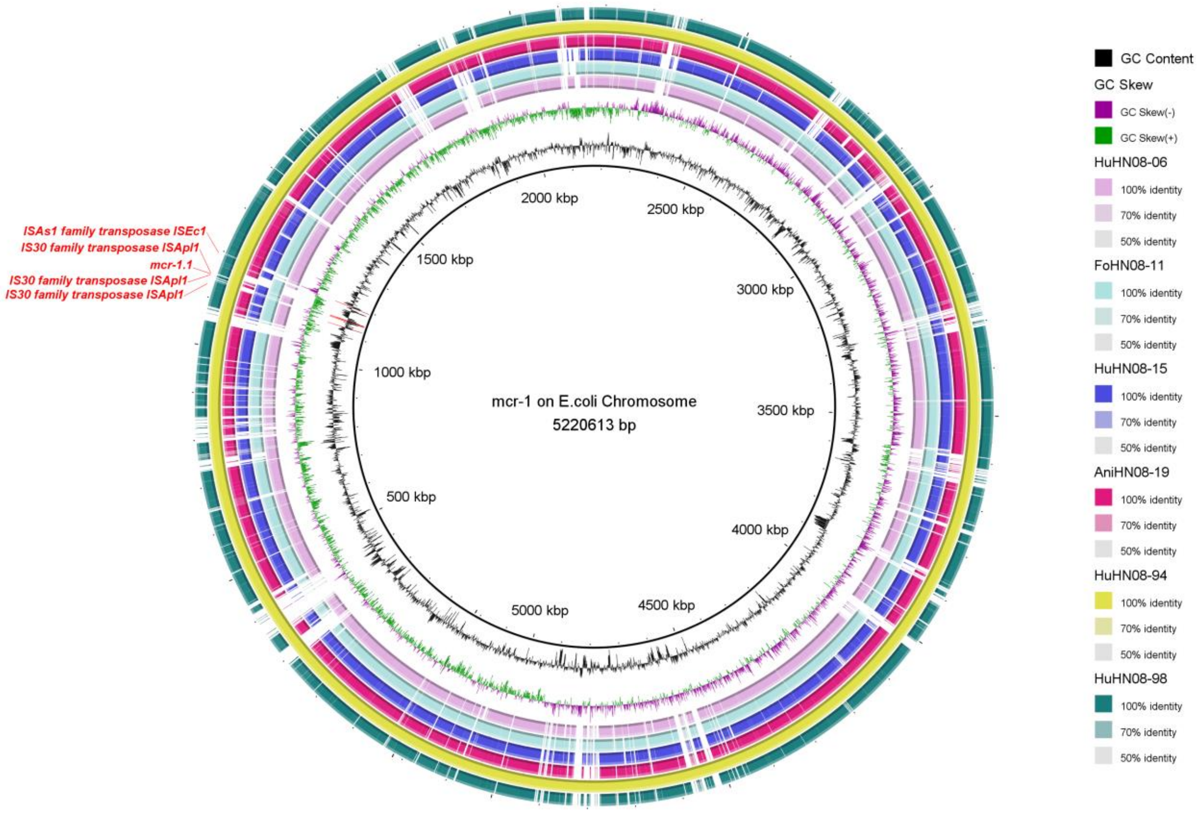

Figure S4: BRIG visualization of draft Mcr1-Ec chromosomes from different origins. The figure show BLAST comparisons against the draft genome sequence of a Mcr1-EC (HuHN08-94) isolated from human faeces in this study. The five rings with different colors correspond to five Mcr1-Ec isolates from human and animal faeces and food, listed in the key. The label in the outer ring represents the annotation of the composite transposon Tn6330 with structure (*ISApl1*-*mcr*-1-*ISApl1*) in this study.

Additional Supplementary Files:

Please see file name: Supplementary.xlsx
